# Glutathione metabolism in type 2 diabetes and its relationship with microvascular complications and glycemia

**DOI:** 10.1371/journal.pone.0198626

**Published:** 2018-06-07

**Authors:** Fallon K. Lutchmansingh, Jean W. Hsu, Franklyn I. Bennett, Asha V. Badaloo, Norma McFarlane-Anderson, Georgiana M. Gordon-Strachan, Rosemarie A. Wright-Pascoe, Farook Jahoor, Michael S. Boyne

**Affiliations:** 1 Department of Basic Medical Sciences, The University of the West Indies, Mona, Jamaica; 2 Children's Nutrition Research Center, Department of Pediatrics, Baylor College of Medicine, Houston, Texas, United States of America; 3 Department of Pathology, University Hospital of the West Indies; Mona, Jamaica; 4 Caribbean Institute for Health Research, The University of the West Indies, Mona, Jamaica; 5 Health Research Unit, Faculty of Medical Sciences, The University of the West Indies, Mona, Jamaica; 6 Department of Medicine, The University of the West Indies, Mona, Jamaica; Universitat de Valencia, SPAIN

## Abstract

**Aims/Hypotheses:**

We hypothesized that there is decreased synthesis of glutathione (GSH) in type 2 diabetes (T2DM) especially in the presence of microvascular complications, and this is dependent on the degree of hyperglycemia.

**Methods:**

In this case-control study, we recruited 16 patients with T2DM (7 without and 9 with microvascular complications), and 8 age- and sex-matched non-diabetic controls. We measured GSH synthesis rate using an infusion of [^2^H_2_]-glycine as isotopic tracer and collection of blood samples for liquid chromatography mass spectrometric analysis.

**Results:**

Compared to the controls, T2DM patients had lower erythrocyte GSH concentrations (0.90 ± 0.42 vs. 0.35 ± 0.30 mmol/L; *P* = 0.001) and absolute synthesis rates (1.03 ± 0.55 vs. 0.50 ± 0.69 mmol/L/day; *P* = 0.01), but not fractional synthesis rates (114 ± 45 vs. 143 ± 82%/day; *P* = 0.07). The magnitudes of changes in patients with complications were greater for both GSH concentrations and absolute synthesis rates (*P-*values ≤ 0.01) compared to controls. There were no differences in GSH concentrations and synthesis rates between T2DM patients with and without complications (*P*-values > 0.1). Fasting glucose and HbA1c did not correlate with GSH concentration or synthesis rates (*P-*values > 0.17).

**Conclusions:**

Compared to non-diabetic controls, patients with T2DM have glutathione deficiency, especially if they have microvascular complications. This is probably due to reduced synthesis and increased irreversible utilization by non-glycemic mechanisms.

## Introduction

The global prevalence of diabetes continues to increase, reaching 8.5% in 2014 [1], thus causing significant mortality and morbidity. Like several developing countries, many patients in the Caribbean with type 2 diabetes display poor control and it is a major public health burden [2, 3]. Chronic hyperglycemia leads to the development of microvascular complications [4, 5] and increases the risk of macrovascular disease [6]. Hyperglycemia-induced cellular damage causes oxidative stress (i.e. increased superoxide production) through a number of mechanisms including increased advanced glycation end products formation, polyol pathway activity, hexosamine activity and protein kinase C activation [5, 7]. Intensive glycemic control decreases the incidence of microvascular complications [4]. Since lowering blood glucose level reduces oxidative stress [8, 9], the reduced risk of microvascular complications may be partially due to improved antioxidant capacity.

Glutathione (γ-glutamyl-cysteinylglycine) is a major intracellular antioxidant and plays a key role in reducing the effects of oxidative stress [10, 11]. Several investigators have reported that erythrocyte glutathione (GSH) concentration is decreased in patients with type 2 diabetes [12–15]. However, the exact mechanisms responsible for glutathione deficiency in type 2 diabetes have not been fully established. We are aware of one study in which GSH synthesis rate was directly measured and those data showed diminished synthesis rates in uncontrolled diabetes [16]. In another study, synthesis although low, was not directly measured, but imputed from the expression of GSH synthetic enzymes [17]. Nevertheless, irreversible utilization may also play a role in reducing GSH concentration. Whereas Afro-Caribbean patients with type 2 diabetes had similar concentrations of total erythrocyte glutathione levels [18] to non-diabetic controls, the concentration of reduced glutathione (GSH) was decreased and oxidized glutathione (GSSG) levels were higher [18] suggesting increased utilization. It is unclear whether impaired glutathione metabolism is a cause or consequence of hyperglycemia although there is some evidence for the later [16]. Although oxidative stress is significantly increased in the presence of microvascular complications [19], it is not clear if patients with complications have more impaired glutathione metabolism compared to diabetic patients without complications.

We hypothesized that there is decreased synthesis of glutathione in type 2 diabetes and this is dependent on the degree of hyperglycemia. Additionally, the abnormal glutathione metabolism is more pronounced in patients with microvascular complications. We measured glutathione concentrations, fractional synthesis rates and absolute synthesis rates in patients with type 2 diabetes, some of whom had microvascular complications, compared to non-diabetic controls using a stable isotope tracer method. We also examined the relationship between glutathione concentration and its synthesis rates with blood glucose concentration and HbA1c.

## Materials and methods

### Study design

In this case-control study, we recruited 16 Afro-Caribbean patients with type 2 diabetes (7 patients without known microvascular complications and 9 with known complications) and 8 non-diabetic controls. Volunteers with type 2 diabetes were recruited from the diabetes specialty clinics at the Diabetes Association of Jamaica and the University Hospital of the West Indies, Kingston, Jamaica. The presence of microvascular complications was determined from their clinic records, i.e. ophthalmologist–diagnosed retinopathy (non-proliferative retinopathy, proliferative retinopathy or a history of laser photocoagulation), nephropathy as evidenced by microalbuminuria (30–300 mcg/day), or physician-diagnosed sensory polyneuropathy (by impaired vibration, crude touch, or monofilament perception). Age- and sex-matched non-diabetic control volunteers were selected after responding to print advertisements. Controls were selected based on having fasting plasma glucose levels <6.1 mmol/L and HbA1c < 6% (42 mmol/mol). Exclusion criteria included major medical illnesses (e.g. cardiovascular disease, cancer, liver or renal failure), morbid obesity (BMI ≥ 40 kg/m^2^) and smoking. We also excluded patients using glargine insulin or thiazolidinediones, since these medications have long durations of action and may affect the degree of oxidative stress on the study day. The Faculty of Medical Sciences/ University Hospital of the West Indies Ethics Committee approved the study protocol. Written informed consent was obtained from all participants.

### Infusion protocol

All participants were asked to abstain from alcohol and severe exercise the day prior to the study. The diabetic participants were asked to withhold their anti-diabetic and any other medications, including statins and ACE–inhibitors, on the day of the study. After an overnight fast, participants were admitted to the metabolic ward of the Tropical Metabolism Research Unit, The University of the West Indies, Mona. Blood pressure and anthropometry were measured using a standardized protocol [20]. Erythrocyte GSH synthesis rate was measured using the precursor/product stable isotope trace technique with intravenous infusion of [^2^H_2_]glycine as tracer and precursor to make GSH (product) according to the methods of Reid and Jahoor [21]. An intravenous line was inserted in each arm for infusion and phlebotomy. Before starting the infusion, blood samples were taken for fasting glucose concentrations, HbA1c, hematocrit, and for baseline isotopic enrichment (2 ml). A priming dose of 20 μmol/kg of [^2^H_2_]glycine was then administered followed by a continuous infusion dose of 15 μmol/kg/h for 8 hours. Additional blood samples (2 ml each) were taken every hour during the last four hours of the infusion. The blood samples were processed and analyzed for isotopic enrichments of RBC-GSH and RBC free glycine, and for RBC-GSH concentration.

### Sample analysis

Immediately after taking each blood sample, two aliquots of 1 mL whole blood were quickly centrifuged at 1,000 *g* for 10 min at 4°C. The packed red cells from one aliquot were immediately stored at -80°C for later analysis of GSH isotopic enrichment. The red cells from the other aliquot were washed thrice with 3 mL of ice-cold sodium chloride solution. The cellular proteins were precipitated using 20% PCA solution and the supernatant was stored at -80°C for analysis of erythrocyte free glycine enrichment and GSH concentration.

### Analysis of isotopic enrichment

To measure the isotopic enrichment of intracellular RBC-GSH, 10 μL of 0.5M dithiothreitol (DTT) solution was added to 100 μL RBC to reduce oxidized glutathione to GSH and its enrichment was measured on a triple quadrupole liquid chromatography mass spectrometer (TSQ Vantage; Thermo Scientific, San Jose, CA). The ions were then analyzed on a Synergi MAX-RP 4 μm 2.0 mm × 150 mm column (Phenomenex) by SRM (selected reaction monitoring) mode. The transitions observed were precursor ions m/z 308 and 310 to product ions m/z 162 and 164, respectively. Instrumental control, data acquisition and analysis were performed by the XCalibur (version 2.1) software package (Thermo Scientific, Waltham, MA).

To measure the isotopic enrichment of intracellular erythrocyte glycine, amino acids were isolated from the RBC-free extract by ion exchange (Dowex 200x) chromatography and converted to the n-propyl ester, heptafluorobutyramide derivative. The isotope ratio was determined by negative chemical ionization gas chromatography-mass spectrometric analysis by selectively monitoring ions at m/z ratios 293 to 295 using a Hewlett Packard 5890 quadrupole mass spectrometer (Hewlett Packard, Palo Alto, CA).

### Measuring GSH concentration

RBC-GSH concentration was measured by an exogenous isotope dilution method using [Gly-^13^C_2_, ^15^N]-GSH (Cambridge Isotope Laboratories) as an internal standard. Briefly, 100 μL of the baseline RBC-free sample was spiked with a known quantity of isotopically labeled [Gly-^13^C_2_, ^15^N]-GSH. The samples were then analyzed by liquid chromatography mass spectrometry as described above. The spiked RBC-GSH was analyzed by selected reaction monitoring at precursor ions m/z 308 and 311 to product ions m/z 162 and 165, respectively.

### Calculations

The fractional synthesis rate (FSR) of erythrocyte GSH was calculated using the formula:

$$\mathrm{FSR}\;(\%/\mathrm{day})=[({\mathrm{IR}}_{t7}–{\mathrm{IR}}_{t5})÷{\mathrm{IR}}_{\mathrm{rbc}}]\times [2400÷(t_{7}–t_{5})]$$

where IRt7—IR_t5_ is the increase in the isotope ratio of RBC-GSH bound glycine between the 5^th^ and 7^th^ hours of infusion, when the isotope ratio of RBC-free glycine, IR_rbc_, had reached a steady state.

The absolute synthesis rate (ASR) of erythrocyte GSH per day was calculated using the formula:

ASR(mmol/L/day)=erythrocyteGSHconcentration×FSR

### Biochemical assays

Fasting glucose concentration was measured by the glucose oxidase method. HbA1c concentration was measured using ion-exchange HPLC techniques with a Bio Rad D-10 machine (Hercules, CA, USA).

### Statistical analysis

Data are expressed as means ± SD. Skewed metabolic variables were log transformed to a normal distribution. Student’s t-test was used to analyze differences between controls and patients with type 2 diabetes. ANOVA and independent-samples Students’ t-tests were used to determine differences in means between the control group and the diabetes group, with and without complications. Age and sex-adjusted multivariate models were also constructed with glutathione metabolism factors as outcome variables. Pearson and Spearman correlations were used to determine associations between the glutathione concentrations, synthetic rates and measures of glycemia (HbA1c and fasting glucose). Data analyses were performed with the SPSS Version 12.0 (Chicago, IL, USA). Results were considered to be statistically significant at *P* < 0.05.

## Results

We approached 120 patients with type 2 diabetes of which there were 4 refusals and 100 had at least one exclusion criterion. Thus, we enrolled 7 patients who had uncomplicated diabetes and 9 who had microvascular complications. In the group with complications, 7 had retinopathy, 7 had neuropathy, and 3 had microalbuminuria, of which 7 had two or more complications. Thirty non-diabetic controls were invited to serve as controls, of which there were 7 refusals, 15 who did not meet matching criteria, resulting in 8 persons who were enrolled in the study. There was no difference in age, BMI, lipids, hemoglobin concentrations and blood pressure between the controls and the diabetic subjects, though two of the diabetic subjects had hypertension (Table 1). As expected, patients with type 2 diabetes had greater waist-hip ratios, higher fasting plasma glucose and marginally higher mean HbA1c than controls.

**Table 1:** Anthropometric and biochemical characteristics of patients with type 2 diabetes (T2DM) with and without microvascular complications, and non-diabetic controls.

	Non-diabetic controls (N = 8)	T2DM and no complications (N = 7)	T2DM and complications (N = 9)	*P*
Age (years)	43.5 ± 8.1	45.3 ± 5.7	50.0 ± 7.2	0.17
% women	50	57	78	0.12
Using insulin (n)	N/A	2	3	-
Using metformin (n)	N/A	6	8	-
Using sulphonylurea (n)	N/A	5	4	-
Using acarbose (n)	N/A	0	2	-
Duration of T2DM (years)	N/A	5.1 ± 4.2	7.1 ± 5.6	0.45
Macrovascular disease (n)	0	0	3	-
BMI (kg/m^2^)	26.2 ± 3.7	31.5 ± 5.5	29.9 ± 6.7	0.07
Waist (cm)	79.5 ± 10.3	95.3 ± 18.7	94.3 ± 18.4	0.12
Waist-hip ratio	0.85 ± 0.07	0.95 ± 0.04	0.90 ± 0.07	0.02
Systolic BP (mm Hg)	133 ± 18	122 ± 7	128 ± 7	0.21
Diastolic BP (mm Hg)	78 ± 7	79 ± 8	80 ± 5	0.81
Fasting glucose (mmol/L)	5.01 ± 0.30	11.09 ± 3.79	8.63 ± 2.79	0.001
HbA1c (%)	5.6 ± 0.4	7.3 ± 1.9	7.7 ± 2.5	0.08
HbA1c (mmol/mol)	38 ± 5	56 ± 20	61 ± 28	0.08
Cholesterol (mmol/L)	4.69 ±0.74	4.74 ± 1.47	4.54 ± 1.05	0.93
HDL-C (mmol/L)	1.37 ± 0.30	1.17 ± 0.33	1.43 ± 0.56	0.45
LDL-C (mmol/L)	3.32 ± 0.83	3.58 ± 1.28	3.11 ± 0.78	0.64
Triglycerides (mmol/L)	1.06 ± 0.84	2.09 ± 2.09	1.27 ± 0.74	0.30
Hemoglobin (g/dL)	13.04 ± 0.84	13.62 ± 1.19	12.91 ± 0.89	0.67

Data are means ± SD. *P* for trend shown.

**Fig 1:**
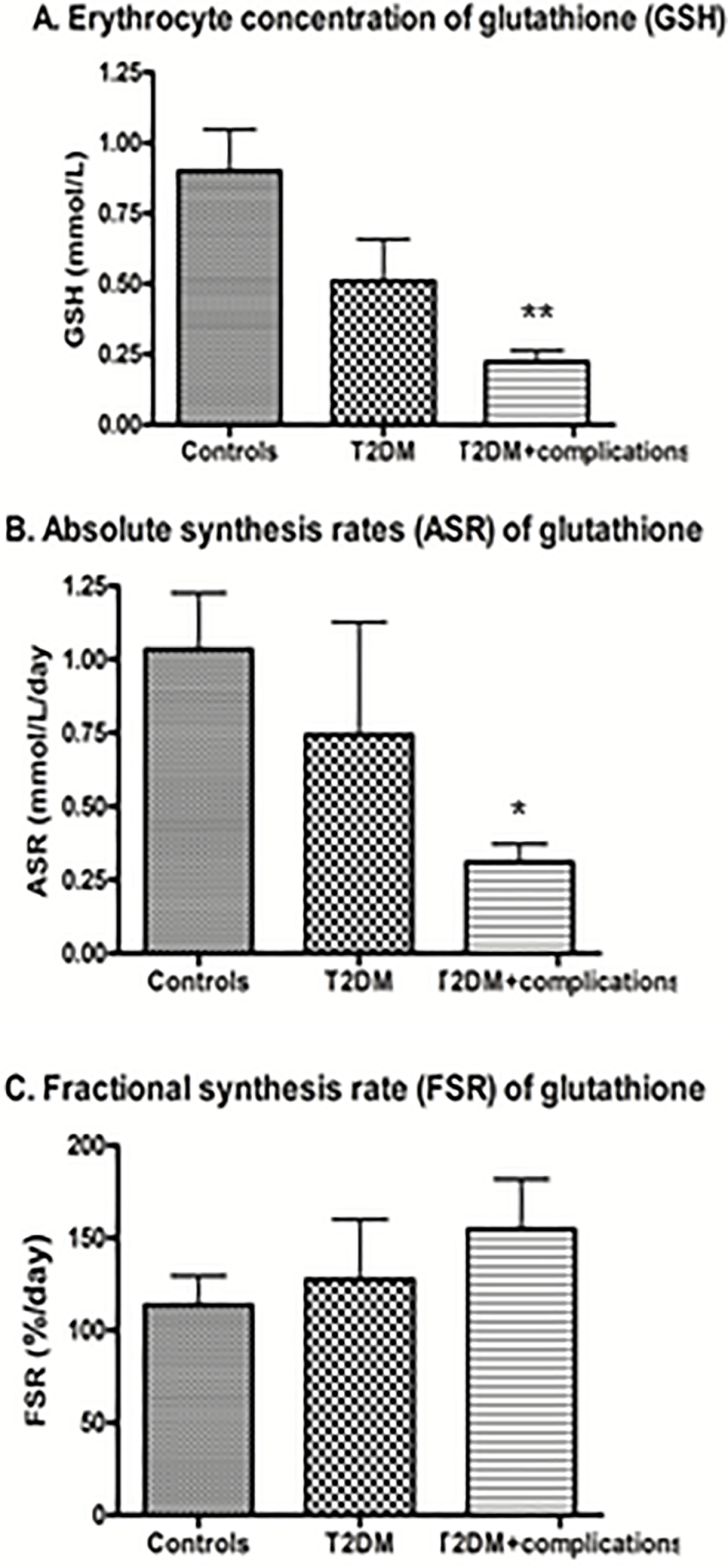
**(A-C). Glutathione metabolism in non-diabetic controls, diabetic patients without microvascular complications, and diabetic patients with complications.** Notes: Data are means ± SD. * P < 0.01 compared to controls; ** *P* < 0.001 compared to controls.

As a group, diabetic patients (combining those with and without complications) had lower GSH concentrations (0.90 ± 0.42 vs. 0.35 ± 0.30 mmol/L; *P* = 0.001) and ASR (1.03 ± 0.55 vs. 0.50 ± 0.69 mmol/L/day; *P* = 0.01), but not FSR (114 ± 45 vs. 143 ± 82%/day; *P* = 0.07) compared to controls. When separated into those with and without complications, glutathione concentrations for diabetic patients without complications trended lower (*P* = 0.06) compared to controls, but absolute synthetic rates did not. Compared to controls, diabetic patients with complications had significantly lower GSH concentrations (*P* < 0.001; Fig 1A) and absolute synthesis rates (*P* = 0.002; Fig 1B), even after adjusting for age and sex. There were no differences in GSH concentrations and absolute synthesis rates between diabetic patients with and without known complications (*P*-values > 0.1). Fractional synthesis rates were not significantly different between controls, diabetic patients and patients with diabetic complications (*P*-values > 0.3) (Fig 1C).

**Fig 2:**
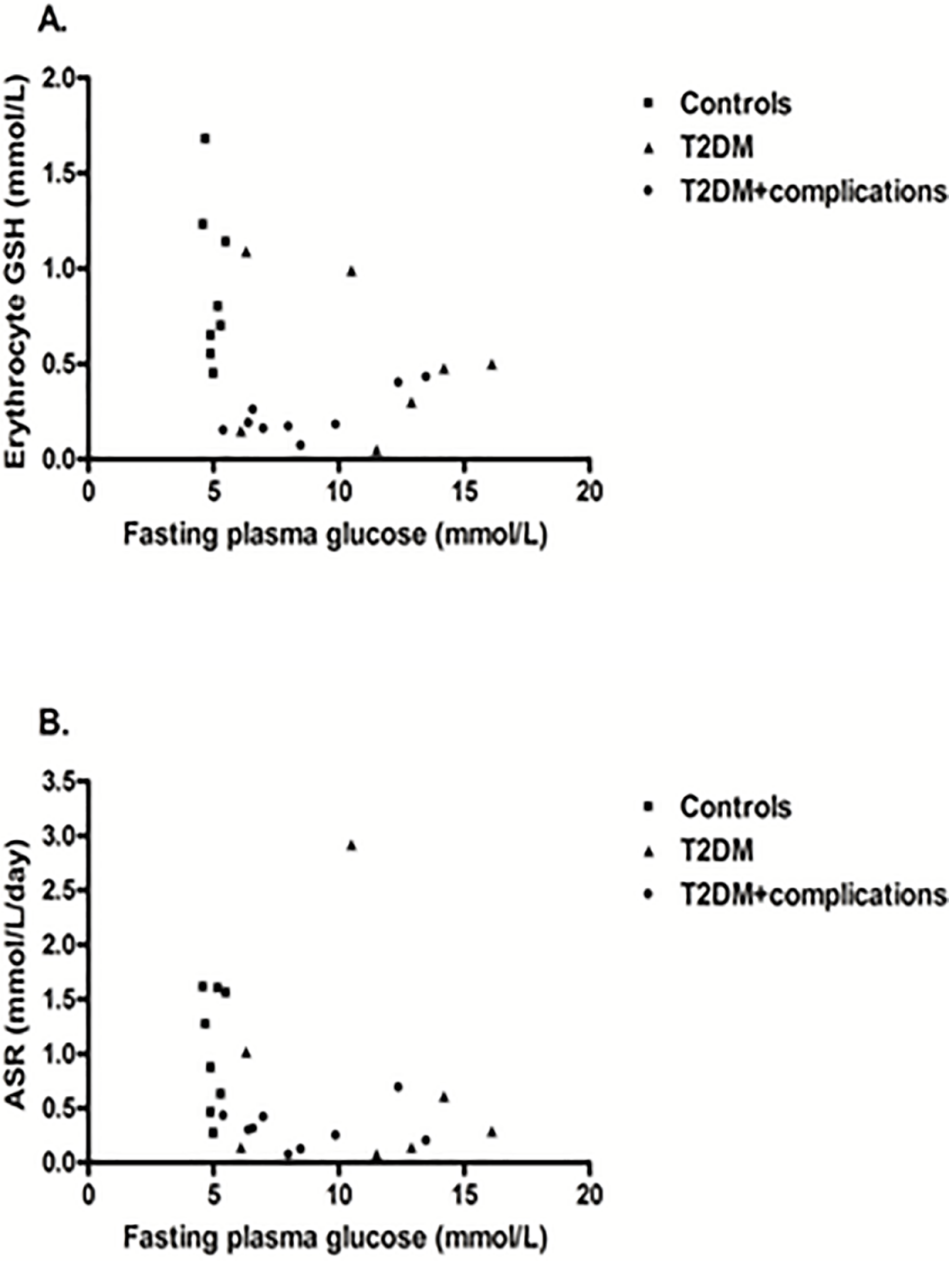
Scatterplots of fasting blood glucose with glutathione concentration in erythrocytes (A) and absolute synthetic rate (B).

**Fig 3:**
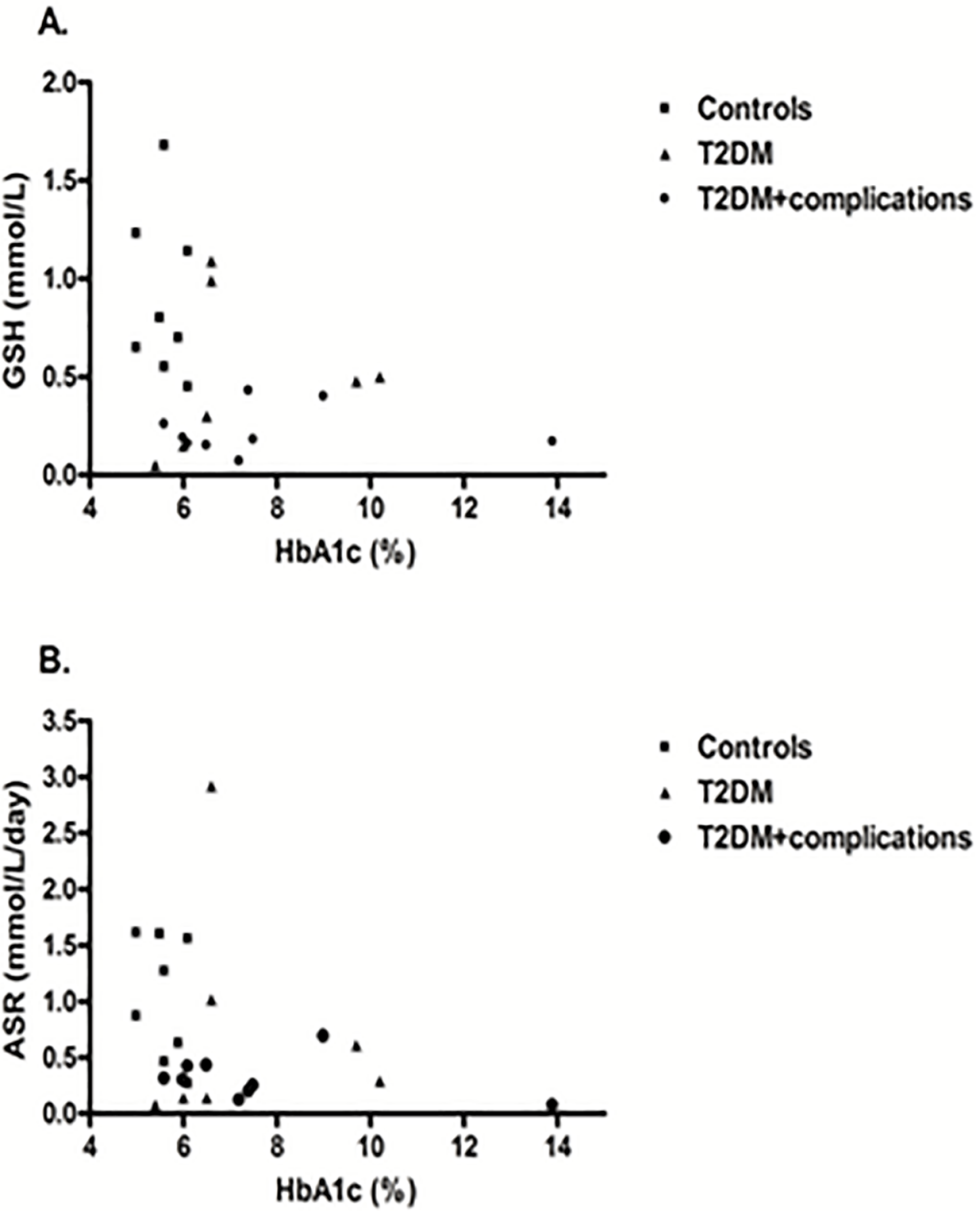
Scatterplots of HbA1c with glutathione concentration in erythrocytes (A) and absolute synthetic rate (B).

GSH concentration was not correlated with fasting glucose (r = -0.32; *P* = 0.12, Fig 2A) or HbA1c (r = -0.25; *P* = 0.26, Fig 3A) even after age and sex adjustment. Also, the absolute and fractional synthesis rates did not correlate with fasting glucose or HbA1c (Figs 2B and 3B; *P*-values > 0.17). These relationships were not different in controls, diabetic patients without complications or with complications. GSH and ASR were not associated with age, any measure of anthropometry (weight, BMI, waist or waist-hip ratio), diastolic blood pressure, hemoglobin, total cholesterol, LDL-C or HDL-C (*P*-values > 0.14). GSH was correlated with systolic blood pressure (r = -0.53; *P* = 0.008), duration of diabetes (r = -0.41; *P* = 0.04) and triglycerides (r = -0.42; *P* = 0.04) but not ASR (*P*-values > 0.14).

## Discussion

Our data showed that type 2 diabetic patients have lower plasma glutathione concentrations and absolute synthesis rates than non-diabetic controls. So, our study, similar to others [12–15, 18, 22, 23] demonstrates glutathione deficiency in type 2 diabetes. However, we additionally observed that glutathione concentrations and absolute synthesis rates were lower in diabetic patients with known microvascular complications compared to controls, an observation not seen in diabetic patients without complications. Our data suggest that reduced GSH synthesis rates and possibly increased irreversible utilization of glutathione contribute to its low concentration in type 2 diabetes. These initial findings should be considered as hypothesis generating and need to be replicated or verified by other investigators.

Glutathione is a tripeptide made from cysteine, glutamate and glycine in two major enzymatic steps [11]. First, glutamyl-cysteine ligase (GCL) catalyzes the formation of gamma-glutamyl cysteine, which is linked to glycine to form GSH by GSH synthetase (GS). Also, extracellular GSH molecule is not transported into cells, but is cleaved into its component amino acids by gamma-glutamyl transpeptidase (GGT) and aminopeptidases. The constituent amino acids are then transported into the cells where they are available to make GSH. Hence, reduced RBC-GSH synthesis may be a consequence of insufficient substrate and/or dysfunction of the enzymes. Reduced concentration of GSH in RBC, plasma and monocytes of individuals with type 2 diabetes are accompanied by diminished expressions of GCL, GS and GGT [17]. Despite these findings, substrate availability may also play a role in reducing GSH synthesis rates, because Sekhar et al showed partial restoration of RBC-GSH concentrations and absolute synthesis rates in uncontrolled type 2 diabetes after cysteine and glycine supplementation [16]. Fractional synthesis rates, which were lower in their diabetic patients, were completely restored with supplementation. Compared to that study, the fractional synthesis rates in our study were not significantly different and were at least 100%/day. It is possible to have a reduction in absolute synthesis rates without a change in fractional synthesis rates because pool size, a determinant of absolute synthesis rates, can vary for the same fractional synthesis rates. We do not know if the pool size of glutathione in the two study populations was different. Also, as shown for proteins, small pools of compounds can turn over more than 100% per day when utilized at increased rates in persons with type 2 diabetes [24, 25] and this may not be affected by insulin therapy. This could represent increased demand for substrates. Improving GSH levels by increasing precursor availability may be important to the metabolic control of diabetic patients since it improved glucose disposal rate and insulin sensitivity, possibly by decreasing free fatty acids and reactive oxygen species as shown in HIV-infected patients [22]. Also, glutathione can prevent increased levels of plasma cytokines induced by acute hyperglycemia [26].

There were no significant correlations between markers of glycaemia (fasting glucose and HbA1c) and glutathione concentrations or absolute synthesis rates. Others have found that acute changes in blood glucose concentration had no appreciable effect on glutathione concentration or fractional synthesis rates in adolescents with poorly controlled type 1 diabetes [23]. This suggests that the changes in glutathione metabolism in diabetes are probably mediated by non-glycemic mechanisms and these mechanisms may be more relevant in persons with microvascular complications. Possible mechanisms could include high levels of non-esterified fatty acids from accelerated lipolysis, oxidative stress propagated by microvascular disease, and pro-inflammatory cytokines [22]. Non-esterified fatty acids are elevated by insulin resistance, and in turn, high levels contribute to the development of insulin resistance. Higher triglycerides were associated with lower glutathione concentration in our data and this may be consistent with an accelerated lipolysis theory. Circulating levels of reactive oxidative species and pro-inflammatory cytokines are elevated in type 2 diabetes, especially if there are microvascular or macrovascular complications which can lower GSH levels [5, 22]. This may support a role of irreversible loss of GSH in reducing GSH concentration in addition to diminished synthesis.

Irreversible utilization of GSH in diabetes may occur with high oxidative stress and increased activity through the polyol pathway. In its role as an antioxidant, GSH is largely oxidized to GSSG in reactions catalyzed by GSH-peroxidase. GSSG is then recycled back to GSH by GSH-reductase using NADPH as cofactor and the ratio of GSH to GSSG regulates redox dependent cell signaling. If the ability to reduce GSSG to GSH is overwhelmed by a high burden of oxidative stress, GSSG can be exported out of cells or react with protein sulphydryl groups to prevent a major shift in the redox equilibrium [11], thereby depleting cellular GSH. Furthermore, although markers of glycemia were not related to GSH, even marginally higher blood glucose, as indicated by HbA1c, may lead to competition for NADPH between GSH-reductase and aldose reductase in the polyol pathway.

Our study has a few limitations. The sample size might have been too modest to detect differences in GSH kinetics between the groups with and without complications. Since this is a cross-sectional observational study, we cannot exclude the possibility of reverse causation, i.e. people with low GSH concentrations or synthesis (perhaps on a genetic basis in some ethnic groups) may have a greater tendency to develop type 2 diabetes and its complications [27–29], although this is controversial [17]. There could also be residual confounding by the lingering effects of anti-diabetic medications such as metformin [30]. Measurement of the enzymes in glutathione metabolism (e.g. GSH-peroxidase, GSH-reductase) would have also been useful. Also, we did not measure oxidative stress, such as isoprostanes (markers of lipid peroxidation) or reactive oxygen species.

We conclude that reduced synthesis contributes to glutathione deficiency in patients with type 2 diabetes, and it is more marked in those with diabetic complications. There may also be increased irreversible utilization of glutathione by non-glycemic mechanisms. The exact mechanisms that affect glutathione metabolism in patients with diabetic complications are unclear and further studies are needed to evaluate the deficiency observed.
